# The Ki-67 proliferation index and recurrence risk of intracranial meningioma: a multicenter, retrospective cohort study of 5,050 patients

**DOI:** 10.1007/s00701-026-06846-y

**Published:** 2026-04-06

**Authors:** Christian Mirian, Lasse Rehné Jensen, Tareq A. Juratli, Andrea Daniela Maier, Anders Broechner, Sverre H. Torp, Helen A. Shih, Ramin A. Morshed, Jacob S. Young, Stephen T. Magill, Luca Bertero, Walter Stummer, Dorothee Cäcilia Spille, Benjamin Brokinkel, Soichi Oya, Satoru Miyawaki, Nobuhito Saito, Martin Proescholdt, Yasuhiro Kuroi, Konstantinos Gousias, Matthias Simon, Jennifer Moliterno, Ricardo Prat-Acin, Stéphane Goutagny, Vikram C. Prabhu, John T. Tsiang, Johannes Wach, Erdem Güresir, Junkoh Yamamoto, Young Zoon Kim, Joo Ho Lee, Daniel W. Kim, Matthew Koshy, Karthikeyan Perumal, Mustafa K. Baskaya, Donald M. Cannon, Dennis C. Shrieve, Chang-Ok Suh, Jong Hee Chang, Maria Kamenova, Sven Straumann, Jehuda Soleman, Ilker Y. Eyüpoglu, Tony Catalan, Austin Lui, Philip V. Theodosopoulos, Michael W. McDermott, Fang Wang, Pedro Góes, Aria Jamshidi, Ricardo Komotar, Michael Ivan, Evan Luther, Luis Souhami, Marie-Christine Guiot, Tamás Csonka, Toshiki Endo, Olivia Claire Barrett, Randy Jensen, Tejpal Gupta, Akash J. Patel, Tiemo J. Klisch, Jun Won Kim, Francesco Maiuri, Valeria Barresi, María Dolores Tabernero, Simon Skyrman, Ian Law, David Scheie, Bjarne Winther Kristensen, Tina Nørgaard Munch, Torstein Meling, Kåre Fugleholm, Paul Blanche, Tiit Mathiesen

**Affiliations:** 1 Department of Neurosurgery, Copenhagen University Hospital, Copenhagen, Denmark; 2 Department of Neurosurgery, Division of Neuro-Oncology, Faculty of Medicine and University Hospital Carl Gustav Carus, Technische Universität Dresden, 01307 Dresden, Germany; 3 Department of Neurosurgery, Laboratory of Translational Neuro-Oncology, Massachusetts General Hospital Cancer Center, Harvard Medical School, Boston, MA USA; 4 Department of Pathology, Bartholin Institute, Rigshospitalet, Copenhagen University Hospital, Copenhagen, Denmark; 5 Department of Clinical and Molecular Medicine, Faculty of Medicine and Health Sciences, Norwegian, University of Science and Technology (NTNU), Laboratory Centre, St. Olavs Hospital, 7491 Trondheim, Norway; 6 Department of Pathology, Laboratory Centre, St. Olavs Hospital, 7030 Trondheim, Norway; 7 Department of Radiation Oncology, Massachusetts General Hospital, Harvard Medical School, Boston, MA USA; 8 Department of Neurological Surgery, University of California San Francisco, San Francisco, CA USA; 9 Department of Neurological Surgery, Feinberg School of Medicine, Northwestern University, Evanston, IL USA; 10 Pathology Unit, Department of Medical Sciences, University and Città Della Salute E Della Scienza University Hospital of Turin, Turin, Italy; 11 Department of Neurosurgery, University of Münster, Münster, Germany; 12 Institute for Neuropathology, University of Münster, Münster, Germany; 13 Deparment of Neurosurgery, Clemenshospital Münster, Münster, Germany; 14 Department of Neurosurgery, Saitama Medical Center/University, Saitama, Japan; 15 Department of Neurosurgery, The University of Tokyo Hospital, Tokyo, Japan; 16 Department of Neurosurgery, University Regensburg Medical Center, Regensburg, Germany; 17 Department of Neurosurgery, Adachi Medical Center, Tokyo Women’s Medical University, Tokyo, Japan; 18 Department of Neurosurgery, Athens Medical Center, Athens, Greece; 19 European University of Cyprus Medical School, Cyprus, Greece; 20 Department of Neurosurgery, Bethel Clinic University of Bielefeld Medical Center, Bielefeld, Germany; 21 Department of Neurosurgery, Yale School of Medicine Yale New Haven Hospital, Smilow Cancer Hospital, New Haven, CT USA; 22 Department of Neurosurgery, Hospital La Fe, Valencia, Spain; 23 Department of Neurosurgery, Université Paris Cité, Beaujon Hospital, Assistance Publique Hôpitaux de Paris, Paris, France; 24 Department of Neurological Surgery, Loyola University Medical Center, Stritch School of Medicine, Maywood, IL USA; 25 Department of Neurosurgery, University Hospital Leipzig, Leipzig, Germany; 26 Department of Neurosurgery, University of Occupational and Environmental Health, Kitakyushu, Japan; 27 Department of Neurosurgery, Samsung Changwon Hospital, Sungkyunkwan University School of Medicine, Changwon, Republic of Korea; 28 Department of Radiation Oncology, Seoul National University College of Medicine, Seoul National University Hospital, Seoul, Republic of Korea; 29 Cancer Research Institute, Seoul National University College of Medicine, Seoul, South Korea; 30 Department of Radiation Oncology, University of Illinois Hospital and Health Sciences System, Chicago, IL USA; 31 Department of Neurosurgery, University of Wisconsin Medical School & Public Health, Madison, WI USA; 32 Department of Radiation Oncology Spencer Fox Eccles School of Medicine, University of Utah, Salt Lake City, UT USA; 33 Department of Radiation Oncology, Yonsei University College of Medicine, Seoul, Republic of Korea; 34 Department of Neurosurgery, Yonsei University College of Medicine, Seoul, Republic of Korea; 35 Department of Neurosurgery, University Hospital Basel, Basel, Switzerland; 36 Division of Neurosurgery, Miami Neuroscience Institute, Miami, FL USA; 37 Department of Neurosurgery, The First Affiliated Hospital of Zhengzhou University, Zhengzhou, Henan China; 38 Department of Neurosurgery, Federal University of São Paulo, São Paulo, Brazil; 39 Department of Neurological Surgery, Sylvester Comprehensive Cancer Center, University of Miami, Coral Gables, FL USA; 40 Department of Neurosurgery, Hackensack Meridian Health, Edison, NJ USA; 41 Department of Neurosurgery, Allegheny General Hospital, Pittsburgh, PA USA; 42 Division of Radiation Oncology, McGill University Health Centre, McGill University, Montreal, QC Canada; 43 Department of Pathology, McGill University Health Centre, Montreal, QC Canada; 44 Department of Pathology, Faculty of Medicine, University of Debrecen, Debrecen, Hungary; 45 Division of Neurosurgery, Tohoku Medical and Pharmaceutical University, Tohoku, Japan; 46 Infirmary Cancer Care, Mobile, AL USA; 47 Department of Neurosurgery, Huntsman Cancer Institute, University of Utah, Salt Lake City, UT USA; 48 Department of Radiation Oncology ACTREC, Tata Memorial Centre, HBNI Kharghar, Navi Mumbai, 410210 India; 49 Department of Neurosurgery, Baylor College of Medicine, Houston, TX USA; 50 Department of Otolaryngology-Head and Neck Surgery, Baylor College of Medicine, Houston, TX USA; 51 Duncan Neurological Research Institute, Texas Children’s Hospital, Houston, TX USA; 52 Department of Molecular and Human Genetics, Baylor College of Medicine, Houston, TX USA; 53 Department of Radiation Oncology, Gangnam Severance Hospital, Yonsei University College of Medicine, Seoul, Republic of Korea; 54 Department of Neurosurgery, University of Naples Federico II, Naples, Italy; 55 Department of Diagnostics and Public Health, University of Verona, Verona, Italy; 56 Unit of Anatomic Pathology- Neuropathology, Fondazione IRCCS Istituto Neurologico Carlo Besta, Milan, Italy; 57 Instituto de Investigación Biomédica de Salamanca (IBSAL), University Hospital of Salamanca, Salamanca, Spain; 58 Department of Clinical Neuroscience, Karolinska Institutet, Stockholm, Sweden; 59 Department of Clinical Physiology and Nuclear Medicine, Copenhagen University Hospital-Rigshospitalet, Copenhagen, Denmark; 60 Department of Clinical Medicine, Faculty of Health and Medical Sciences, University of Copenhagen, Copenhagen, Denmark; 61 Department of Clinical Medicine and Biotech Research and Innovation Center (BRIC), University of Copenhagen, Copenhagen, Denmark; 62 Depatrment of Congenital Disorders, Statens Serum Institut, Copenhagen, Denmark; 63 Department of Neurological Surgery, Istituto Nazionale Neurologico “C.Besta”, Milan, Italy; 64 Section of Biostatistics, Department of Public Health, University of Copenhagen, Copenhagen, Denmark

**Keywords:** Meningioma, Recurrence, Ki-67, Simpson Grade, Risk, Prediction

## Abstract

**Purpose:**

The Ki-67 proliferation index (Ki-67 PI) has been associated with meningioma recurrence, yet its clinical utility remains debated. Whether Ki-67 PI provides prognostic information across subgroups defined by both WHO grade and extent of resection remains to be investigated.

**Methods:**

We analyzed 5,050 patients with intracranial meningiomas from the international *PERNS* cohort (42 centers, diagnosed between 1989–2019) who underwent surgical resection without postoperative radiotherapy. Ki-67 PI prognostic accuracy was assessed up to 10 years postoperatively by using ROC analyses and estimating its association with the risk of recurrence.

**Results:**

Results demonstrated that the prognostic value of Ki-67 PI differed by subgroups defined by WHO grade and Simpson grade. For patients with the same Simpson grade (1–3), the predictive accuracy of Ki-67 PI for 10-year recurrence risk was stronger in WHO-2 than in WHO-1. Within WHO-1 and WHO-2 meningiomas, the predictive accuracy of Ki-67 PI increased with higher Simpson grade (1–3). However, no predictive value was observed in Simpson grade 4 resections regardless of WHO grade.

**Conclusion:**

These findings highlight that Ki-67 PI should be interpreted in the context of both WHO grade and extent of resection, and, if done so, may offer potential value to refine individualized surveillance strategies in meningioma patients with gross total resection in the initial 10-year postoperative timeframe. Findings cannot be extrapolated beyond 10 years, which may be particularly relevant for WHO-1 tumors with low Ki-67 PI and Simpson grade 1 resection.

**Supplementary Information:**

The online version contains supplementary material available at 10.1007/s00701-026-06846-y.

## Introduction

Meningiomas represent a wide and heterogeneous tumor category, and subgrouping is essential to capture differences in biology and prognosis. Traditionally, this has been achieved through WHO grading, and more recently refined by integrating molecular biomarkers such as *1p/22q* co-deletion, isolated *1p* loss or *1q* gain, *TERT* promoter mutations, and homozygous *CDKN2A/B* deletions [18, 20, 24, 25, 37, 39]. Molecular classification of meningiomas has advanced over the past decade, from early genomic studies of recurrent mutations and copy number alterations to methylation-based systems that show promise for improving risk stratification of meningiomas [4, 9–12, 14, 27, 28, 30, 31, 38]. However, the most recent molecular classification systems are not yet routinely implemented in clinical practice and do not currently consider how more general parameters such as the extent of resection may influence their association with recurrence or progression [10, 27, 28, 35, 37, 38, 44]. Therefore, the histological evaluation of tumor samples remains integral to define the WHO grade of meningiomas, with a specific focus on histological features such as mitotic rate and brain invasion [20, 37].

In this context, the Ki-67 proliferation index (Ki-67 PI) is a prognostic biomarker universally used for prognostication of various tumor types since more than three decades, with its expression increasing as cells progress through the proliferative phases of the cell cycle [5, 17, 41, 42]. In meningiomas, the Ki-67 PI is positively correlated with WHO grade and has been associated with recurrence in numerous studies, yet its clinical utility remains debated. Meta-analyses aggregating these studies show significant heterogeneity, reflecting considerable variation in the reported strength of association between Ki-67 PI and recurrence [1, 3, 19]. Many of the underlying studies are limited by small sample sizes, heterogenous follow-up, rely on univariable analyses or multivariable analysis adjusted differently. A further limitation is the absence of analyses specifically evaluating the prognostic role of Ki-67 PI in relation to extent of resection. Although some studies have adjusted for resection in multivariable models, none have assessed whether its prognostic use depends on WHO grade and extent of resection [1, 3, 19].

Whether Ki-67 PI provides prognostic value in detecting recurrence across subgroups defined by WHO grade and Simpson grade remains uncertain. To address this, the aim of the study was to analyze the association between Ki-67 PI and 10-year recurrence risk across these subgroups, using data from a large, international multicenter cohort of patients with primary meningiomas.

## Methods

### Database and study design

The *PERNS* (PERsonalized NeuroSurgery) database is a multicenter, international, retrospective database including adult patients who underwent resection of an intracranial meningioma and includes 7,992 patients collected from 42 different institutions. The *PERNS* database reflects a large and demographically diverse group of patients with meningioma who were diagnosed, treated, and followed between 1989 and 2019. Clinical and histopathological data review was performed locally by representative physicians from various departments, including neurosurgery, neuropathology, neurology, and radiation oncology.

**Fig. 1:**
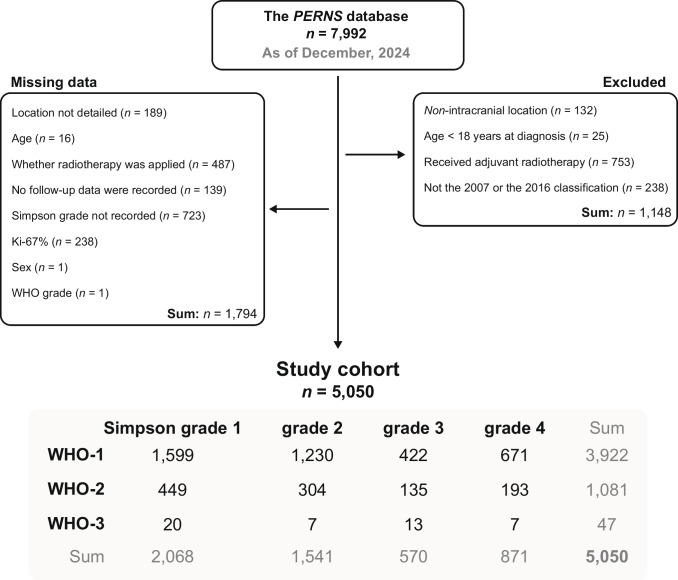
Flow chart of generating the study population using our international *PERNS* database

From the *PERNS* database, eligible patients for this study were 18.0 years or older at diagnosis and were classified based on the contemporary WHO classification at the time of surgery (2007 or 2016 edition of the WHO classification). Information on Simpson grade was required for the study objective of analyzing Ki-67 PI in relation to extent of resection. Patients were excluded if they receive any form of adjuvant radiation in the postoperative period, regardless of WHO grade. Evaluating the prognostic role of Ki-67 PI in patients receiving radiotherapy was considered a related but distinct research question beyond the scope of this study. Patients with missing data were excluded (Fig. 1). Missing values mainly reflect that each participating institution contributed data based on its specific clinical practices and research focus, meaning that certain variables were recorded in some cohorts but not universally across all centers.

### Outcomes

The main endpoints were the 5 and 10-year cumulative incidence of recurrence, accounting for the competing risk of recurrence-free death, in patients treated surgically without adjuvant radiotherapy. Follow-up was defined as the time since surgery, with each patient tracked until recurrence detected, recurrence-free death, or last clinical follow-up recorded within 10 years postoperatively. Recurrence after gross total resection (Simpson grades 1–3) or progression of residual tumor after subtotal resection (Simpson grade 4) was defined and recorded locally, according to standard practices within each center. Because no standardized follow-up guidelines exist, recurrence/progression data may be influenced by differences in follow-up intensity across centers and time periods. However, patients were followed at specialized centers with multidisciplinary teams performing MRI and/or CT scans systematically. While "progression" may be a more accurate term for patients with subtotal resection (Simpson grade 4), we use the term "recurrence" throughout for consistency and practicality. We did not have information on how Ki-67 PI was quantified (hot-spot or whole-slide; digital or manual), which is acknowledged as a limitation.

### Statistical analysis

We employed two analytical strategies. First, we performed subgroup analyses stratified by WHO grade and Simpson grade. Here the aim was to quantify the added value of Ki-67 PI to the WHO and Simpson grade for the prognosis of recurrence, without relying on parametric modeling assumptions. Second, we used multivariable regression models to estimate the association between Ki-67 PI and recurrence risk adjusted for additional predictors of recurrence. Here the aim was to quantify the added value of Ki-67 to more predictors of recurrence than only WHO and Simpson grade, using parametric modeling assumptions.

1. Subgroup analysis

The prognostic value of Ki-67 PI was evaluated using ROC curves within subgroups of WHO grade and Simpson grade. Within these subgroups, the accuracy of Ki-67 PI in predicting the 10-years risk of recurrence was summarized using area under the ROC curve (AUC) [7]. To define the ROC curve, cases were the patients experiencing a recurrence within 10 years, whereas controls were those who did not. We stratified patients into low- and high-risk categories using a 4% Ki-67 PI cut-off, as commonly used in prior literature to dichotomize risk groups, and estimated the corresponding positive (PPV) and negative predictive values (NPV) [1, 3, 19]. We used inverse probability of censoring weighting to account for censoring and the competing risk of recurrence-free death [7].

For each WHO grade, separate logistic regression models were fitted to estimate the association between Ki-67 PI and recurrence risk at 5 and 10 years postoperatively using inverse probability of censoring weighting, again to account for censoring and competing risks [8]. We used cubic splines to fit a flexible model which could capture both linear and non-linear associations. The location of the knots was chosen to match the different distributions of Ki-67 PI within each WHO grade subgroup. For comparison, we added the population-average recurrence risk (referred to as the “marginal-risk”) at 5- and 10-years postoperatively using the Aalen-Johansen estimator (i.e., unadjusted estimates) [26].

Moreover, subgroups were defined using Ki-67 PI quantiles within each WHO grade. For each subgroup, the Aalen-Johansen estimator was used to estimate the recurrence risk within 10 years. Subsequently, we further stratified by Simpson grade and computed the area under each Aalen-Johansen curve. This provides estimates of mean time to recurrence within 10 years. Specifically, this “mean time” derived as the area under the curve should be interpreted as the mean time to recurrence for patients who experienced a recurrence within 10 years postoperatively, or time to 10 years, for those who did not experience a recurrence. This approach properly accounts for both loss to follow-up within 10 years (right-censoring) and competing risks, and does not correspond to simply averaging the observed times [6, 45].

(2) Multivariable regression analysis

Finally, we quantified the association between Ki-67 PI and recurrence risk adjusted for more predictors of recurrence than only WHO and Simpson grade, which was based on the intraoperative assessment by the neurosurgeon and not routinely verified by postoperative imaging [40]. We report adjusted odds ratio that quantify the change in 10-year risk of recurrence when comparing the risk between two patients who differ by 1%-point in Ki-67 PI, but who are otherwise similar with respect to all predictor variables adjusted for: sex (male/female), age (categorized as < 50, 50–70, or > 70 years), Simpson grade, Ki-67 PI, intracranial location (with 10 levels: (1) Convexity, (2) Cerebellopontine Angle/Cerebellar Convexity/Tentorium/Petrous, (3) Intraventricular, (4) Lateral Fossa Anterior, (5) Optic, (6) Parasagittal/falx, (7) Planum Sphenoidale/Olfactory Groove/Clinoid, (8) Spheno-Orbital/Sphenoid Medial Wing/Petroclival, (9) Sphenoid Lateral Wing, and (10) Tuberculum Sella/Clivus/Foramen Magnum/Sinus Cavernosus), and year of diagnosis to account for time varying practices (categorically: < 2001, 2001–2006, and $\geq$2007). To estimate the association between Ki-67 PI and recurrence within each Simpson grade, and to investigate whether this relationship varied across grades, an interaction term between Ki-67 PI and Simpson grade was included. The adjusted odds ratios were estimated and compared between Simpson grades using a logistic regression model fitted via inverse probability of censoring weighting. The Kaplan–Meier method stratified on aforementioned “year of diagnosis”-groups was used to compute the weights [8].

## Results

Out of 7,992 patients in the *PERNS* database, the study cohort comprised 5,050 patients following the inclusion and exclusion criteria (Fig. 1). The study cohort was followed for a total of 24,503 person-years with a median follow-up of 4.3 years (range: 0.1 to 28.7 years). During follow-up, 724 recurrences and 290 recurrence-free deaths were recorded. We summarized baseline characteristics stratified by WHO grade (Table 1).

**Table 1:** Baseline characteristics of patients with meningiomas, stratified by WHO grade. Values are presented as counts with percentages unless otherwise stated

Covariate	WHO-1 (*n* = 3,922)	WHO-2 (*n* = 1,081)	WHO-3 (*n* = 47)
Ki-67 PI (%) Median (IQR)	2 (1, 4)	7 (5, 10)	21 (15, 30)
Simpson grade			
1	1,599 (41%)	449 (42%)	20 (43%)
2	1,230 (31%)	304 (28%)	7 (15%)
3	422 (11%)	135 (12%)	13 (28%)
4	671 (17%)	193 (18%)	7 (15%)
Age group			
< 50 years	1,041 (27%)	268 (25%)	13 (28%)
50–70 years	2,097 (53%)	544 (50%)	21 (45%)
> 70 years	784 (20%)	269 (25%)	13 (28%)
Sex			
Female	2,925 (75%)	645 (60%)	25 (53%)
Male	997 (25%)	436 (40%)	22 (47%)
Calendar year of diagnosis			
Before 2001	286 (7%)	98 (9%)	3 (6%)
Before 2007	689 (18%)	173 (16%)	7 (15%)
In or after 2007	2,490 (63%)	750 (69%)	16 (34%)
Missing*	457 (12%)	60 (6%)	21 (45%)
WHO classification			
2007 edition	2,817 (72%)	566 (52%)	23 (49%)
2016 edition	1,105 (28%)	515 (48%)	24 (51%)
Marginal risk of recurrence**			
5-years	7.6% (6.4–8.7)	22.7% (19.5–25.9)	56.5% (40.9–72.0)
10-years post-surgery	14.7% (12.6 −16.7)	34.6% (30.3–39.0)	61.0% (44.9–77.1)

* Missing values were primarily due to data protection regulations that restrict the use of dates

** Marginal risks (with 95% CI) were estimated using data from patients with Simpson grade 1–3

### Subgroup analysis

**Fig. 2:**
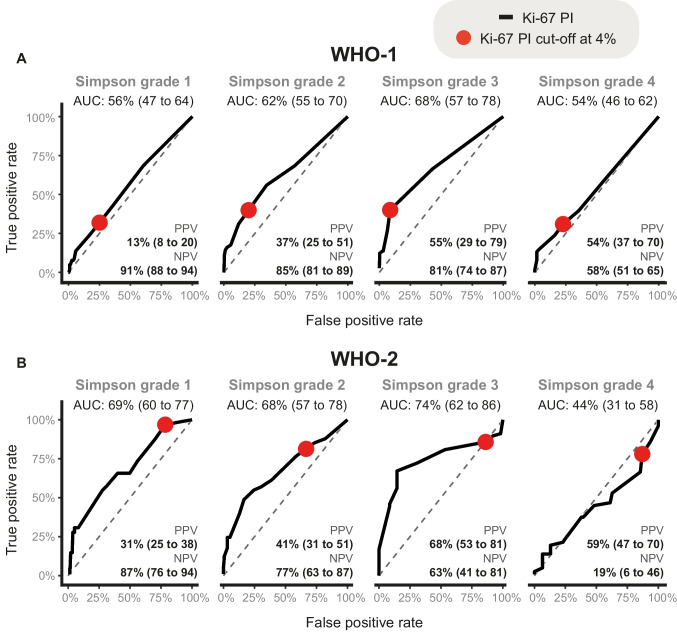
Prognostic value of Ki-67 PI in detecting recurrence 10-year post-surgery, evaluated using ROC curves and AUC metrics. The analysis was stratified by subgroups of Simpson grade within WHO-1 and grade 2 meningiomas, whereas PPV and NPV derived from the 4% cut-off (red dot). **A** WHO-1 and (**B**) WHO-2 meningiomas

The initial ROC analyses focused on Simpson grade 1–3 resections with AUC with 95% CIs reported in Fig. 2. For both WHO-1 and WHO-2 meningiomas, the results suggest that the higher the Simpson grade of resection, the greater the predictive accuracy of Ki-67 PI for the 10-year risk of recurrence. When comparing WHO-1 and WHO-2 within the same Simpson grade, Ki-67 consistently showed stronger predictive accuracy with 10-year recurrence in WHO-2, indicating that its prognostic value rises with increasing tumor grade from WHO-1 to WHO-2 (WHO-3 had too few observations for meaningful analysis). In contrast, for Simpson grade 4 resections, Ki-67 PI did not appear to be informative for predicting 10-year recurrence in either WHO-1 or WHO-2 tumors, suggesting limited utility for subtotal resection in the *PERNS* cohort (Fig. 2).

We subsequently evaluated the predictive accuracy of a prognostic test that stratified patients into “high” or “low” risk of 10-year recurrence based on a Ki-67 PI cut-off of 4%. This categorization did not substantially improve risk stratification for WHO-1 with Simpson grade 1 (marginal risk: 9.6%, PPV using the cut-off: 13.0%). However, we found significantly improved predictive accuracy for Simpson grade 2 (marginal risk: 18.3%, PPV: 37.0%) and Simpson grade 3 (marginal risk: 22.8%, PPV: 55.1%); i.e., more than doubling the estimated risk when comparing the risk of a patient with Ki-67 PI above 4% to that of a random patient of the same subgroup (Fig. 2). For WHO-2, incorporating the 4% Ki-67 PI cut-off only provided minimal additional information when comparing its PPV with the marginal risks: Simpson grade 1 (marginal risk: 26.7%, PPV: 31.2%), Simpson grade 2 (marginal risk: 34.5%, PPV: 40.8%) and Simpson grade 3 (marginal risk: 58.8%, PPV: 68.4%) (Fig. 2).

### Exclusion of simpson grade 4

Based on the results described above, we focused subsequent analyses on patients who underwent Simpson grade 1–3 resections (i.e. gross total resection), where the association between Ki-67 PI and recurrence appeared most clinically relevant within the *PERNS* cohort. A total of 871 patients with a Simpson grade 4 resection were excluded, yielding a study cohort of 4,179 patients for further analysis (Table 1). The cumulative follow-up time was 20,571 person-years, with a median individual follow-up of 4.4 years (range: 0.1 to 28.7 years).

**Fig. 3:**
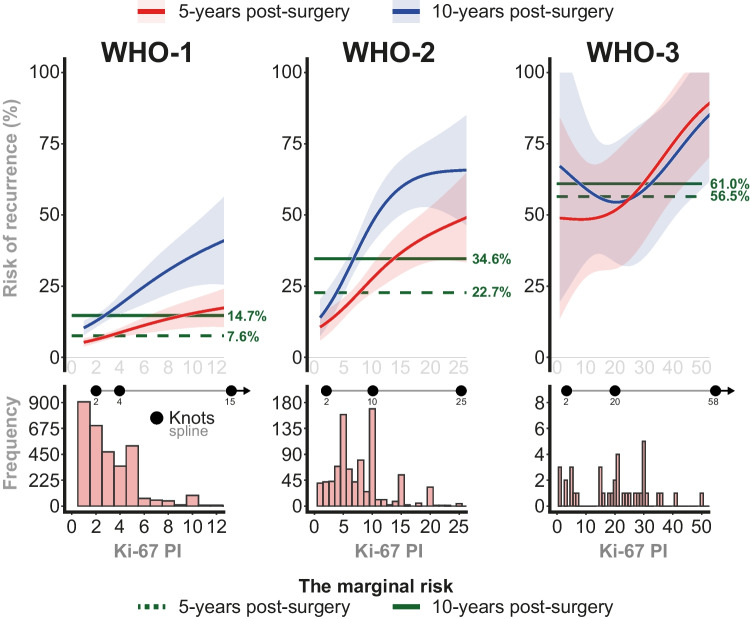
**A** Ki-67 PI risk of recurrence for individual WHO grades at 5- and 10-years postoperatively. Flexible modeling by using splines with internal knots located depending on the subgroup specific distribution of Ki-67 PI which is represented by the histogram below the curves (**B**). The marginal risks, i.e. the unadjusted, population-average risk of recurrence, are shown for each WHO grade

In this subcohort of 4,179 patients, we assessed the association between Ki-67 PI and recurrence risk separately for each WHO meningioma grade. For WHO-1, both the 5- and 10-year recurrence risks increased gradually as Ki-67 PIs rose from 2 to 12%, where the recurrence risks exceeded the marginal risks at both the 5- and 10-year time points once Ki-67 PIs surpassed 3% (Fig. 3). For WHO-2, the 5-year recurrence risk showed a gradual increase with Ki-67 PIs ranging from 2 to 25%, exceeding the marginal risk (22.7%) at a Ki-67 PI of 8%. In contrast, the 10-year recurrence risk plateaued at ~ 65.0% for Ki-67 PIs$\geq$17%, while the marginal risk (34.6%) was exceeded at a Ki-67 PI of 7% (Fig. 3). For WHO-3, a similar trend could not be identified, because of the limited power resulting from a small sample size.

**Fig. 4:**
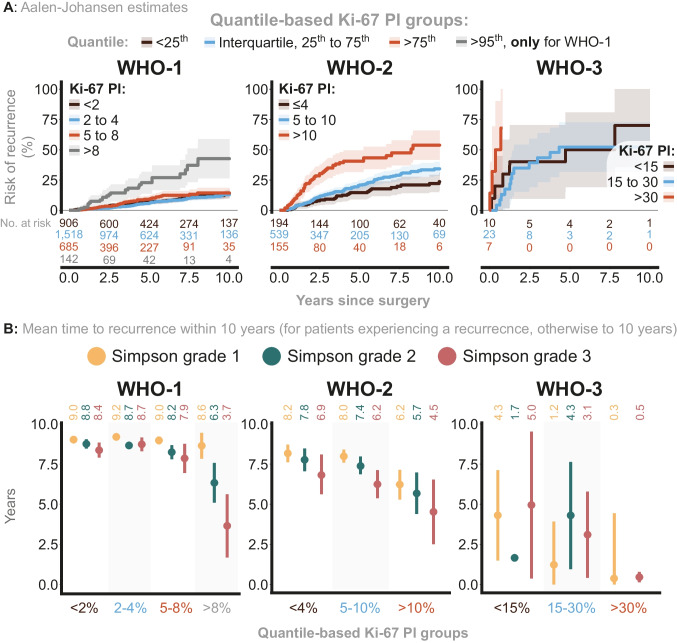
**A** Risk of recurrence estimated using the Aalen-Johansen method. WHO-1 patients were categorized into four groups based on Ki-67 PI: below the 25th percentile, within the interquartile range (25th to 75th percentile), above the 75th percentile, and finally the 95th percentile. For WHO-2 and WHO-3, three Ki-67 PI groups were defined: below the 25th percentile, within the interquartile range, and above the 75th percentile. **B** Within each WHO grade, the quantile-based Ki-67 PI groups were further stratified on Simpson grade. For these subgroups, we report the mean time to recurrence within 10 years for patients that experienced a recurrence, or otherwise 10 years

Finally, we used quantile-based Ki-67 PI groups within each WHO grade (with numbers at risk shown in Fig. 4). For WHO-1, no difference in recurrence risk was observed between following quantile-based Ki-67 PI groups: < 2% (below the 25th percentile), 2–4% (25th to 75th percentile), and 4–8% (above the 75th percentile) (Fig. 4A). In addition, the large sample size enabled us to consider an additional “upper 5%”-category for Ki-67 PI, which corresponded to > 8%. In this “upper 5%”-subgroup, the 10-year recurrence risk was 42.7% (95% CI: 26 to 59), considerably contrasting the remaining quantile-based groups which showed 10-year recurrence risks between 13.0% and 14.3%. WHO-2 demonstrated a clear risk difference across the quantile-based Ki-67 PI groups: < 4% (below the 25th percentile), 5–10% (25th to 75th percentile), and > 10% (above the 75th percentile) (Fig. 4A). The 10-year recurrence risk for WHO-2 with Ki-67 PI > 10% was 53.8% (95% CI: 41.7 to 65.9). The small sample size of WHO-3 patients led to large uncertainty that prevented the drawing of meaningful conclusions. However, the quantile-based groups were: < 15% (below the 25th percentile), 15–30% (25th to 75th percentile), and > 30% (above the 75th percentile) (Fig. 4A).

Subsequently, we estimated, for each subgroup stratified by WHO grade, Simpson grade, and Ki-67 PI group, the mean time to recurrence within 10 years (specifically, the mean time to either recurrence, when it occurred within 10 years, or 10 years, otherwise, as described above).

The mean time to recurrence for the quantile-based Ki-67 PI groups varied across the Simpson grades, particularly for WHO-1 with Ki-67 PI > 8% (Fig. 4B). Specifically, those who underwent a Simpson grade 3 resection had a mean time to recurrence of 3.7 years (95% CI: 1.7 to 5.6) while their Simpson grade 1 counterpart showed a mean time to recurrence of 8.6 years (95% CI: 7.8 to 9.4). For WHO-2, both higher quantile-based Ki-67 PI groups and higher Simpson grades were associated with a shorter mean time to recurrence. For WHO-3 patients, the small sample size introduced uncertainty that prevented meaningful conclusions from being drawn (Fig. 4B).

### Multivariable regression analysis

**Fig. 5:**
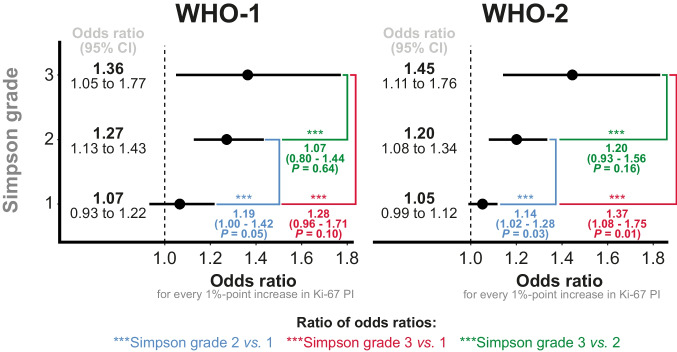
Ki-67 PI and its association with risk of recurrence depending Simpson grade and WHO grade. The analysis was adjusted for age group (< 50, 50–70, > 70), sex, Simpson grade, Ki-67 PI, the year of diagnosis (< 2001, 2001–2006, and $\geq$2007), and intracranial locations

Finally, focusing on WHO-1 and WHO-2 meningiomas, we found that the association between Ki-67 PI and 10-year recurrence risk varied across Simpson grades. This association was found increasingly stronger with increasing Simpson grade (Fig. 5). These results align well with those of the ROC analysis already presented above in Fig. 2; the further adjustment did not change the main conclusions.

### Post-hoc analysis

We performed a post-hoc analysis to further explore the “upper 5%” WHO-1 category with a Ki-67 PI > 8%, as those presented in Fig. 4A were not stratified on Simpson grade. Therefore, stratifying further on Simpson grade, post-hoc Aalen-Johansen estimates were obtained to examine the 5-year recurrence risks of the WHO-1 quantile-based Ki-67 PI groups (shown in Supplementary Fig. 1, including numbers at risk). The three lower Ki-67 PI groups (< 2%, 2–4%, and 5–8%) exhibited similar recurrence risks within each Simpson grade, whereas Ki-67 PI > 8% was associated with progressively worse outcomes across increasing Simpson grades (supporting that the Aalen-Johansen estimates in Fig. 4A were not markedly confounded by Simpson grade).

## Discussion

We found in this retrospective multicenter study of 5,050 primary meningiomas treated with surgery alone that Ki-67 PI should be interpreted in relation to both WHO grade and Simpson grade as its prognostic value is not uniform across these subgroups.

For patients who had the same Simpson grade, the association between Ki-67 PI and recurrence risk was stronger in WHO-2 than in WHO-1 patients. However, our analyses can only reflect recurrences detected within the initial 10 years and cannot be extrapolated beyond this timeframe. The median follow-up of *PERNS* cohort was 4.3 years, reflecting a follow-up that corresponds to an interval where a larger proportion of recurrences would be expected in WHO-2 compared to WHO-1 meningiomas [21, 22, 32]. Specifically, no prognostic benefit was observed from incorporating Ki-67 PI into WHO-1 patients who underwent a Simpson grade 1 resection. This may reflect truly radical resections, but benign tumor biology and small residuals might also prolong recurrence-free survival beyond 10 years. The present findings should therefore be interpreted within the *PERNS* cohort and the 10-year timeframe; after this period, the association between Ki-67 PI and recurrence—particularly in WHO-1 with Simpson grade 1 resections—may be different for patients followed for longer time.

Among patients who underwent gross total resection and had the same WHO grade (either grade 1 or grade 2), increasing Simpson grade was associated with a stronger association between recurrence risk and Ki-67 PI. Similarly, the likelihood of detecting recurrences within the 10-year follow-up increases with higher Simpson grades, whereas smaller residuals may recur later and cannot be extrapolated from the present analysis [2, 15]. In contrast, Ki-67 PI did not affect the predictive ability after subtotal Simpson grade 4 resection and could possibly reflect the high risk of recurrence associated with a subtotal resection or surveillance bias, as patients with known residual tumor are typically followed more closely. In addition, patients treated with adjuvant radiotherapy were excluded, potentially introducing selection bias, as this therapy is typically administered in patients with more high-risk features.

### Ki-67 PI as biomarker to refine follow-up strategies

A key finding was the strong association between mean time to recurrence and subgroups defined by quantile-based Ki-67 PI, Simpson grade, and WHO grade (WHO-1 and WHO-2). These results suggest that combining Ki-67 PI with extent of resection may offer a practical opportunity to tailor postoperative surveillance more precisely, although further validation is warranted. For instance, patients with high Ki-67 PI and (microscopic) subtotal resection may benefit from intensified early follow-up, whereas those with low Ki-67 PI and gross total resection could potentially avoid unnecessary imaging and clinic visits. As an example, WHO-1 patients with both high Ki-67 PI and high Simpson grade may require close monitoring in the early years, while those with low Ki-67 PI and low Simpson grade might not need reassessment for several years. Overall, the Ki-67 PI remains an attractive biomarker for refining patient-tailored surveillance, particularly given its widespread use in pathology, which facilitates validation across large cohorts and integration into existing clinical workflows.

### The 4% Ki-67 PI cut-off

The predictive values of Ki-67 PIs were different across WHO and Simpson grades. While the 4% cut-off showed limited applicability to WHO-2 meningiomas, it demonstrated some utility in WHO-1 patients with Simpson grade 2 and 3 resections by identifying more than twice as many recurrences within 10 years relative to the marginal risk (the unadjusted, population-average risk).

More generally, a cut-off value that helps clinicians distinguish between low- and high-risk patients is an appealing tool for guiding treatment and follow-up strategies. However, the evaluation of such cut-off values must go beyond individual-level risk prediction and consider their broader implications for clinical practice at the healthcare system level. Cut-off values inevitably result in misclassification—false positives and false negatives—which carry different consequences for both patients and the healthcare system. False negatives (patients incorrectly classified as low-risk despite being high-risk) may delay necessary interventions and should be considered more critical to avoid. However, minimizing false negatives often comes at the cost of increasing false positives (patients incorrectly labeled high-risk), which may lead to unnecessary monitoring, patient anxiety, and ultimately increase the burden on clinical resources. As healthcare systems vary in structure and capacity, the acceptable balance between these trade-offs is inherently context-dependent. Therefore, defining an “optimal” cut-off requires not only statistical validity but also careful consideration of its practical feasibility and impact on diverse healthcare systems – thus, recognizing that a universal cut-off value may not be achievable. Therefore, using Ki-67 PI cut-off values are not recommended.

### Strengths and limitations

The inclusion of data from 5,050 patients with a primary meningioma across 42 institutions comprising a comprehensive dataset of large-scale real-world variability. Thus, exploration of potentially different subgroups is enabled, leading to a more nuanced risk stratification in analyzing the association between recurrence risk and Ki-67 PI.

A key limitation is selection bias, inherent to the non-standardized inclusion process of the *PERNS* database, where participating institutions contributed data based on local research and clinical priorities. This variability could lead to differences in patient selection (countries, calendar time, treatment protocols), tumor characteristics (such as molecular properties or histological features), and follow-up protocols across centers. The majority of patients were diagnosed and followed before standardized guidelines for radiological follow-up—such as Response Assessment in Neuro-Oncology (RANO) criteria [16]; hence variation in surveillance intensity across centers and eras may influence the timing of detected recurrences, potentially biasing time-to-event estimates toward shorter recurrence times in more intensively followed patients. We focused on surgically treated patients who did not receive radiotherapy, as the association between Ki-67 PI and recurrence in irradiated patients was considered a separate research question beyond the scope of this present study. The choice of 5- and 10-year horizons reflects clinically meaningful follow-up intervals and represents a balance between long-term risk assessment and available follow-up support within this large, multicenter cohort. In addition, the cohort was still meaningfully sized at 10 years (*n* = 312 for WHO-1 and *n* = 115 for WHO-2, Fig. 4); however, it is acknowledged that WHO-1 might experience a recurrence beyond this timeframe. Moreover, the Ki-67 PI is not a fully standardized metric and has limited inter- and intra-observer reproducibility reported across tumor types [23, 29, 33, 34, 43]. Manual counting includes subjectively selected areas to evaluate with a possible focus on hot spots. It has been investigated whether automated and digital counting may provide more objective and reproducible approaches to assess Ki-67 PI. Digital methods for Ki-67 PI quantification in meningioma, including whole-slide image analysis and automated hot-spot selection, which have demonstrated strong correlations with manual assessments and improved interobserver agreement. Whole-slide image analysis which provides a quantitative measure of overall proliferative activity, whereas hot-spot selection focuses on the most proliferative areas within a tumor [23, 29, 33, 34, 36, 43]. The variability associated with differences Ki-67 PI quantification methods may also be evident in our data. In the histogram (Fig. 3B), prominent peaks at Ki-67 PI-values divisible by five (5, 10, 15, 20, etc.) likely indicate that pathologists frequently rounded or approximated values. In this study, we lacked detailed information on how Ki-67 PI was assessed, potentially introducing bias to an unknown extent.

The lack of molecular profiling limits adjustment to important molecular biomarkers. However, the vast majority of patients was followed prior to implementation of molecular biomarkers and was therefore unavailable. Moreover, we used data classified according to either the 2007 or 2016 WHO editions. The only relevant change is the introduction of brain invasion as a standalone criterion for WHO grade 2 meningioma, which affected only 3.9% of cases in a previous study, and is therefore considered a minor limitation [36].

## Conclusions

Our findings highlight that Ki-67 PI should be interpreted alongside extent of resection for optimal prognostic utility (Simpson grade and within WHO-1 and WHO-2), as its predictive value is not uniform across these subgroups. However, Ki-67 PI appeared to provide valuable information and could be utilized to better individualize patient follow-up when combined with as such. WHO-1 meningiomas with Ki-67 PI > 8% exhibited recurrence risks and mean time to recurrence similar to WHO-2 and WHO-3 cases. We cannot extrapolate risk of recurrence beyond 10 years, which has particular relevance for WHO-1 meningiomas with Simpson grade 1 and low Ki-67 PI.

## Supplementary Information

Below is the link to the electronic supplementary material.

ESM 1 Supplementary Material 1: Aalen-Johansen estimates of the 5-year recurrence risk for quantile-based Ki-67 PI groups, stratified by WHO grade and Simpson grade. The 5-year timepoint was chosen due to reduced sample sizes beyond that timepoint. (PDF 58.1 KB)

## Data Availability

Data not available due to ethical and legal restrictions.
