# Supplementary figures and images for: The Ki-67 proliferation index and recurrence risk of intracranial meningioma: a multicenter, retrospective cohort study of 5,050 patients
Source: Acta Neurochir (Wien). 2026 Apr 6;168(1):124. doi: 10.1007/s00701-026-06846-y (PMC13230318; doi:10.1007/s00701-026-06846-y)

# WHO-1

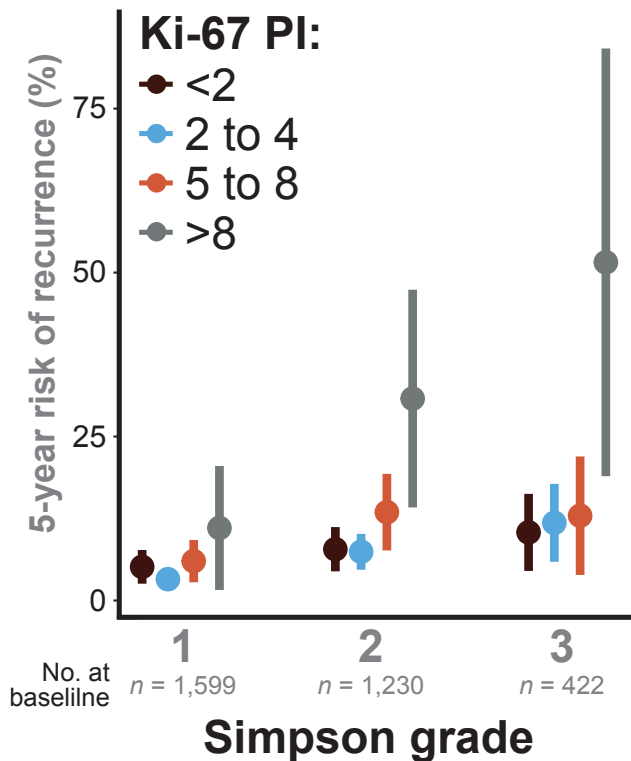

# WHO-2

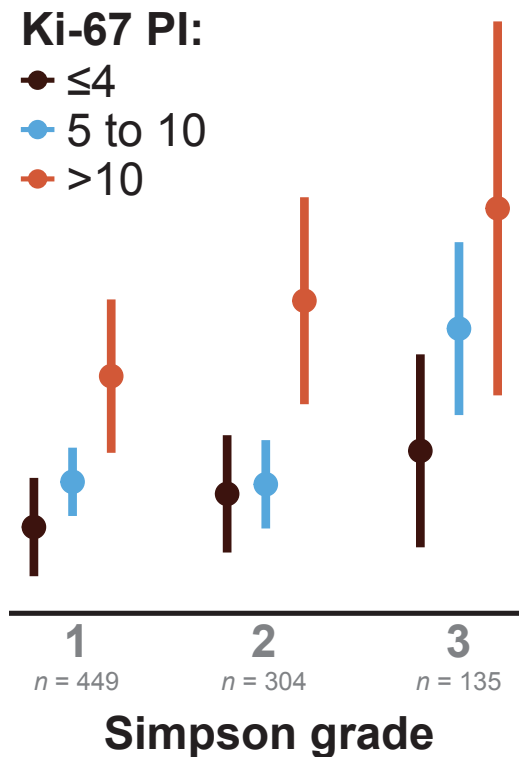

Supplement: Supplementary file 1 — Supplementary Material 1: Aalen-Johansen estimates of the 5-year recurrence risk for quantile-based Ki-67 PI groups, stratified by WHO grade and Simpson grade. The 5-year timepoint was chosen due to reduced sample sizes beyond that timepoint. (PDF 58.1 KB) [file 701_2026_6846_MOESM1_ESM.pdf]
